# The *Staphylococcus aureus* RNome and Its Commitment to Virulence

**DOI:** 10.1371/journal.ppat.1002006

**Published:** 2011-03-10

**Authors:** Brice Felden, François Vandenesch, Philippe Bouloc, Pascale Romby

**Affiliations:** 1 Université de Rennes, Inserm U835, UPRES EA2311, Biochimie Pharmaceutique, Rennes, France; 2 Inserm U851, Université de Lyon, Centre National de Référence des Staphylocoques, Lyon, France; 3 Laboratoire Signalisation et Réseaux de Régulations Bactériens, Institut de Génétique et Microbiologie, CNRS, UMR 8621, IFR115, Centre Scientifique d'Orsay, Université Paris Sud 11, Orsay, France; 4 Architecture et Réactivité de l'ARN, Université de Strasbourg, CNRS, IBMC, Strasbourg, France; University of California San Diego, United States of America

## Abstract

*Staphylococcus aureus* is a major human pathogen causing a wide spectrum of nosocomial and community-associated infections with high morbidity and mortality. *S. aureus* generates a large number of virulence factors whose timing and expression levels are precisely tuned by regulatory proteins and RNAs. The aptitude of bacteria to use RNAs to rapidly modify gene expression, including virulence factors in response to stress or environmental changes, and to survive in a host is an evolving concept. Here, we focus on the recently inventoried *S. aureus* regulatory RNAs, with emphasis on those with identified functions, two of which are directly involved in pathogenicity.

## Introduction

*Staphylococcus aureus* belongs to the normal human flora. About one in three healthy individuals are colonized asymptomatically with *S. aureus* in the nostrils without any associated disease. However, *S. aureus* is also a leading cause of hospital- and community-acquired infections worldwide [1]. This potent Gram-positive pathogen can grow in any part of the human body, and also propagates in other animals. The severity and locations of infections vary widely, from minor skin infections to deep-seated infections such as endocarditis, bone and joint infections, or severe pneumonia. Concern about *S. aureu*s infections is heightened because of the emergence and spread of hypervirulent, drug-resistant, and community-acquired strains [2]. The pathogenesis of *S. aureus* is intricate and relies on an arsenal of virulence-associated factors including toxins, adhesins, enzymes, and immune-modulators [3]. These proteins are delivered in a coordinated manner by sophisticated regulatory networks. To this end, multiple *trans*-acting modulators, including regulatory proteins, secondary metabolites, small peptides, and RNAs, are brought into play [4], [5].

**Figure 1:**
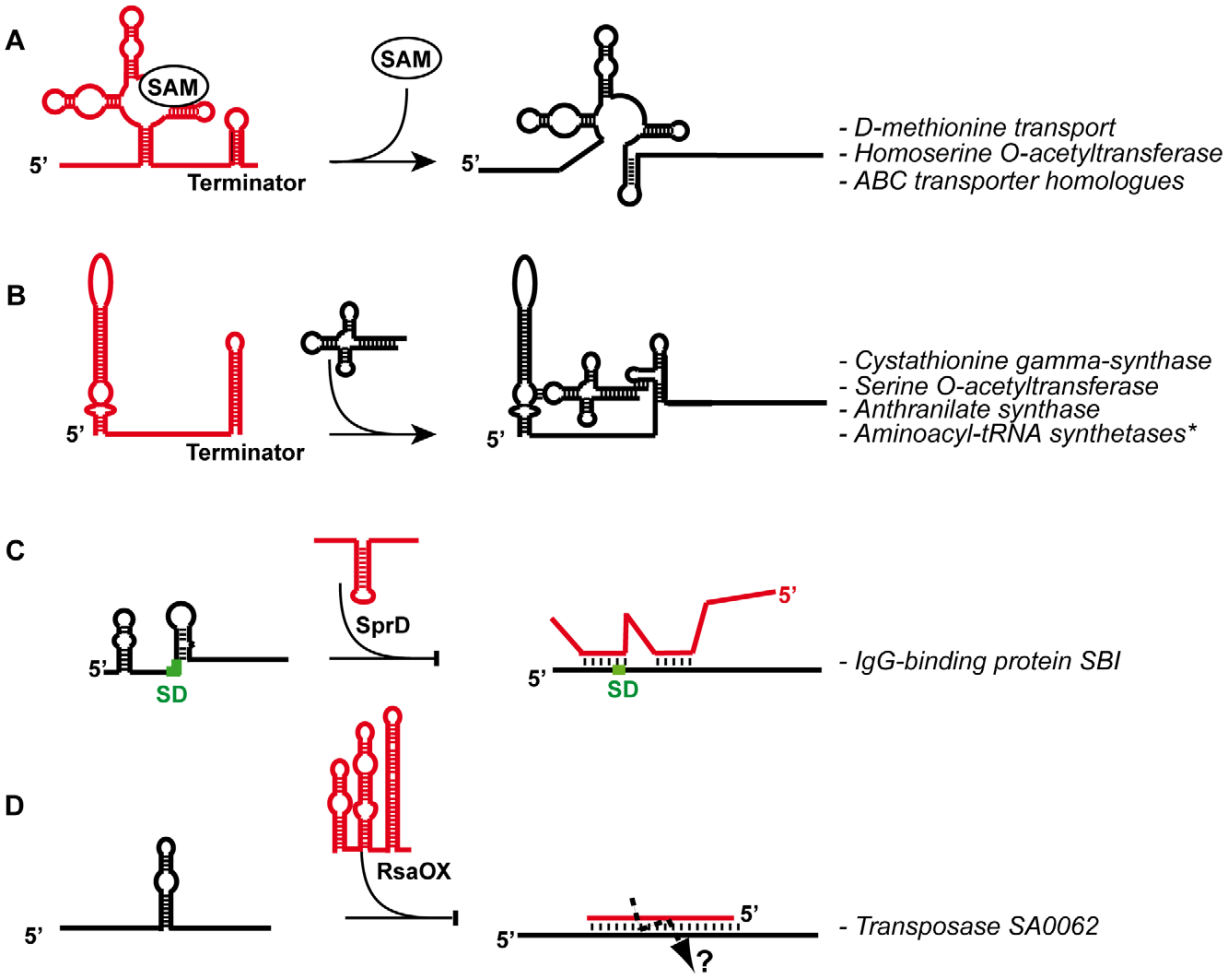
General mechanisms given for several *S. aureus* regulatory RNAs. (A) S-adenosyl methionine (SAM) riboswitch regulates several operons encoding enzymes and transporter proteins. SAM binds to an aptamer domain and stabilizes the formation of a terminator hairpin (the alternate pairings are in red) to arrest transcription [75]. (B) Schematic representation of a T-Box involved in the regulation of aminoacyl-tRNA synthetases (aaRS). Non aminoacylated tRNA binds to the leader region at two different sites: the anticodon sequence of the tRNA base paired with a codon-like triplet present in the “specifier loop”, and the ACCA end of the tRNA binds to a complementary sequence located in the T-Box motif [82]. This interaction stabilizes an anti-terminator structure allowing transcription of the downstream genes. *The aminoacyl-tRNA synthetases regulated by this mechanism are the ValRS, MetRS, IleRS, PheRS, GlyRS, SerRS, HisRS, and the AspRS. (C) The SprD pathogenicity island RNA (in red) binds to the ribosome binding site (The SD is green) of *sbi* mRNA to repress translation initiation [37]. (D) The RsaOX (in red) (or Teg14as) *cis*-acting asRNA [17], [18] is complementary to the coding sequence of *tnp* mRNA and is predicted to induce rapid degradation of *tnp* mRNA. Both the asRNA and the mRNA target site are highly folded, suggesting that the pairing is initiated by a loop–loop interaction.

The universality of small, usually non-coding, RNAs (sRNAs) playing a role in gene regulation in bacteria is now well established [6], [7]. The number of sRNA identified in bacteria has considerably increased this past decade [8]. Most of them exert regulatory functions by interacting with proteins and by pairing with mRNAs. Besides these *trans*-acting sRNAs, a variety of large mRNA leaders sense environmental cues or intracellular concentrations of small metabolites to adopt structures that prevent/activate their extended transcription or translation. Examples of sRNA-dependent regulations are given in Figure 1. Recent studies on various bacteria indicated that pervasive transcription generates massive antisense transcription [9]–[11]. All these sRNAs are members of regulatory circuits involved in metabolism, stress adaptation, and virulence. Although still a recent field, the study of sRNAs has already extended our knowledge of regulatory circuits in bacteria, in relation to pathogenesis. In *S. aureus*, the multifunctional regulatory RNA, called RNAIII, is a paradigm for RNA-mediated regulation of virulence genes [12], [13]. It is the effector of the accessory gene regulator (*agr*) system [4], which controls the switch between the expression of surface proteins and excreted toxins. Within the last few years, several reports highlighted the importance and diversity of staphylococcal sRNAs [14]–[18]. This review focuses on *S. aureus* regulatory RNAs including RNAIII, newly discovered island-encoded sRNAs, *cis*-encoded antisense RNAs (asRNA), and *cis*-acting regulatory regions of mRNAs. For all these RNAs, their structural diversity and phylogenetic distribution is documented and discussed, with emphasis on those for which their targets were identified and regulatory mechanisms elucidated. Some of these sRNAs have been demonstrated as tractable targets for compounds inhibiting *S. aureus* pathogenesis.

## Diversity of sRNAs Expressed from the *S. aureus* Genome

The complex structure of RNAIII, the first sRNA reported in *S. aureus*, and the intriguing pleiotropic phenotypes associated with its inactivation, led to the proposal and subsequent demonstration that RNAIII was a regulatory RNA [12] (cf. below). In 2001, with the exception of tmRNA, the sRNAs were ignored from the analysis of the first staphylococcal genome sequences [19]. As sRNAs initially emerged as major regulators for bacterial physiology in *Escherichia coli*, several laboratories engaged in a quest to identify sRNAs in various *S. aureus* strains. In 2005, Pichon and Felden demonstrated for the first time the existence of sRNAs produced by horizontally acquired genomic islands by identifying seven sRNAs encoded on pathogenicity islands (PIs) in *S. aureus* [14]. Recently, several publications on this bacterium have contributed to an impressive catalog of putative and experimentally validated sRNAs that place *S. aureus* as a new model organism for sRNA studies. Approaches for identifications were based on dedicated computing software [14], [15], [20], Affymetrix microarrays [21], [22], conventional cloning/sequencing of small sized cDNAs [16], and 454 [17] and Illumina [18] high throughput sequencing (HTS). The sRNA genes are located randomly in the core genome and mobile accessory elements, and some of them are present in multiple copies. Besides the housekeeping RNAs (such as 4.5S, RNase P, and tmRNA), 6S RNA, and *cis*-acting regulatory sequences, conservation of most sRNAs is restricted to the genus *Staphylococcus*, and among them, about 50% are found so far only within the *S. aureus* species. Approximately 100 *trans*-encoded sRNAs, 100 *cis*-encoded asRNAs, and more than 30 *cis*-acting regulatory regions of mRNAs (e.g., riboswitch, T-Box, protein-binding motif) were discovered to be encoded on the *S. aureus* chromosome, and nine sRNAs on the pN315 plasmid. The expression of more than 90 of these was confirmed by alternative methods such as northern blots, RNA extremity mapping, or RT-qPCR (Table S1). The HTS study performed by Beaume et al. [18] confirmed almost all sRNAs from other studies [14], [15], [17], [20], with the exception of 12 sRNAs that were reported solely by Abu-Qatouseh et al. [16]. This singularity might reflect the distance between the unsequenced clinical isolates and the *S. aureus* strains in which sRNAs are mainly studied. This observation may suggest that the sRNA profile is a signature of a given strain; if in the case of N315 we are approaching a full inventory, it is not the case for the other *S. aureus* strains.

### Numerous *cis*-Encoded Antisense RNAs (asRNAs)

**Figure 2:**
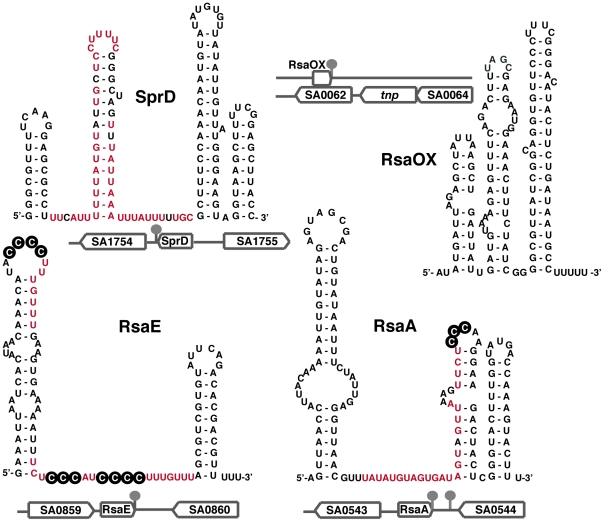
Secondary structures of selected *S. aureus* sRNAs. The secondary structures of RsaA and RsaE [15] and of SprD [37] were experimentally determined. The secondary structure of the antisense RNA RsaOX (or Teg14as) is proposed based on computer predictions [17], [18]. The genomic locations and flanking genes of the sRNAs are indicated. The known regulatory domains of the sRNA, which bind to mRNA targets, are in red. The black circled nucleotides are accessible C-rich motifs that are proposed to be crucial for the initial binding with mRNA [15].

These RNAs pair with an extended perfect match to RNAs expressed from their complementary gene strand (Figure 1D). The first one identified in *S. aureus* was shown to control the rolling-circle replication of plasmid pT181 by transcriptional attenuation [23]; the striking discovery of the recent studies is the large proportion of asRNAs among the inventoried sRNAs [14], [16]–[18]. Many asRNAs are expressed from PIs and mobile elements (plasmids or transposons). Transposable genetic elements are important motors of genetic variability but can also compromise genome integrity. Hence, transposition would expectedly be tightly regulated. The control of transposase synthesis occurs through different mechanisms, one being by asRNAs [24]. Among them, RsaOX is complementary to the coding sequence of SA0062 mRNA encoding a transposase [17] (Figure 1D). Another interesting case is the control of the IS1181 transposase, which has its gene repeated eight times in the *S. aureus* N315 genome. Two small RNAs, Teg17/RsaOW and Teg24as complementary to the 5′ and 3′ IS1181 UTRs, respectively, were detected. The expression of Teg17/RsaOW is constitutive during growth [17], and is strongly enhanced in response to pH and temperature changes [18]. Interestingly, these asRNAs (Figures 1D and 2) show predicted structural similarity to the *E. coli* “RNA-OUT” asRNA, which regulates *tnp* translation of the IS10 insertion element, suggesting that these asRNAs have been tuned for fast binding to mRNAs [25]–[27]. Some of these asRNAs are surprisingly long; for example, one of them, which is complementary to SA0620 encoding a secretory antigen, SsaA-like, exceeds 1 kb [18]. AsRNAs may participate in the differential expression of genes belonging to the same operon; this could be the case for two asRNAs that are complementary to *capF* and *capM* mRNA regions of the large *cap* operon mRNA encoding enzyme for capsular polysaccharide synthesis [16], [18]. Several overlapping 3′UTRs of convergent mRNAs were also detected in staphylococci, in which the 3′UTR of one mRNA overlaps the mRNA on the opposite strand, and convergent genes share the same terminator hairpin. Since they pair between each other with extended perfect matches, these 3′UTRs could be categorized as asRNAs. sRNAs that likely issue from the processing of extended 3′ UTRs were also found. How these overlapping and processed UTRs affect gene expression is unknown [18].

### A Global sRNA Expression Variation Associated with a Phenotype

Small colony variant (SCV) isolates exhibit particular properties such as host intracellular persistence and the ability to cause antibiotic-refractory, latent, or recurrent infections [28]. Several asRNAs and sRNAs showed differential expression in the SCV compared to a wild type strain [16]. In addition, SCVs does not express RNAIII [16], [29]. SCVs also repressed an asRNA that presumably regulates expression of PhoB, an alkaline phosphatase involved in inorganic ion transport [16]. These variations of regulatory RNA expression may correlate with electron transport deficiencies associated with SCVs [28]. Hence, perturbation of genetic regulatory circuits and their associated effects on virulence may be a consequence of and/or contribute to the SCV phenotype.

### RNome-Related *S. aureus* Specificities

*S. aureus* has a small genome (2.8 M bp) with a low GC composition (32.8%); these features affect its RNome and it is likely that the features of sRNA as learned from studies in enteric bacteria will differ in *S. aureus*. Specifically, in *E. coli* and many other bacteria, RNase E is implicated in the sRNA-dependent mRNA turnover, and the RNA chaperone Hfq is required for the activity of most *trans*-encoded sRNAs and, as shown recently, for a *cis*-encoded asRNA [27]. However, *S. aureus* do not have an RNase E, but instead possesses RNases J1, J2, and Y functional homologs [30]. In *Bacillus subtilis*, a complex resembling the *E. coli* degradosome, including the three RNases J1/J2/Y, glycolytic enzymes (enolase, phosphofructokinase PfkA) and the RNA helicase CshA were recently reported [31]–[33]. Homologs of all these enzymes are present in the staphylococcal strains, but whether these enzymes form a “degradosome” remains to be addressed [30]. Concerning Hfq, this protein is not expressed in several tested *S. aureus* strains [34], [35], and the deletion of its corresponding gene was thought to have no effect on bacterial physiology [36]. However, a recent report indicates that in strains where Hfq is detected, the deletion of its coding gene could result in decreased toxicity and virulence of *S. aureus*, leading to the conclusion that Hfq is a global regulator that controls pathogenicity [35]. In these strains, analyzing more precisely the regulatory functions of Hfq as well as its involvement on the characterized sRNA-dependent regulations would be required. The commitment of Hfq is not straightforward since several strains in which Hfq is not detected produce toxins and are virulent. Moreover, Hfq is not required for the reported cases of sRNA-induced translational repression [13], [15], [34], [37]; in several of these examples, the sRNAs efficiently bind to the mRNA targets and form extended pairings that are specifically cleaved by the double-strand-specific RNase III [13], [38], [39]. The dispensability of Hfq in several *S. aureus* strains could be due, as compared to *E. coli*, to the presence of longer “sRNA–mRNA” hybrids that compensate for low GC content of the pairings [40].

General strategies for the use of RNA-dependent regulation by bacteria vary according to species as the result of environmental and evolutionary constraints. For instance, in *B. subtilis* and *S. aureus*, the autocatalytic site-specific cleavage in the 5′ UTR of *glmS* mRNA, encoding glucosamine 6 phosphate synthase, is stimulated by the binding of glucosamine-6-phosphate; in *E. coli* and *Salmonella* the *glmS* gene is regulated by two sRNAs [41]–[43]. Mechanistic and functional analyses performed on several *S. aureus* sRNAs revealed its RNome specificity by pointing out its differential roles in the regulation of mobile elements, metabolism, stress adaptation, and virulence.

## Pathogenicity Island–Encoded RNAs

Mobile genetic elements have essential roles in genome evolution. In facultative pathogens such as *S. aureus*, they mediate acquisition of antibiotic resistance genes, including the highly problematic methicillin resistance via the staphylococcal chromosome *mec* cassette (SCCmec), and have conferred a wide range of adaptive processes for survival in their hosts. Among these elements, which include phages, genomic islands, transposons, and plasmids, the horizontally acquired PIs are the repository of many toxins, adherence and invasion factors, superantigens, and secretion systems [44], [45]. In addition to the protein-coding genes, several SaPIs including phage-related chromosomal islands encode and express several sRNAs [14], [16]–[18] (Table 1). Some sRNAs are present in multiple copies scattered around the *S. aureus* genome (up to eight genomic copies; repeated events of horizontal transfer as well as gene duplications may account for the presence of multiple copies), and additional copies are on plasmids [14]. The location of sRNAs on SaPIs suggests that these sRNAs would play important roles during *S. aureus* infections.

**Table 1:** RNAs expressed from N315 *S. aureus* PIs.

RNAs	Flanking Genes	Strand Orientation	Locations	Lengths^a^	Ends Mapping^a^	Exp. Validation	References
**SaPIn1**							
Teg21as	*Antisens to hypot. protein SA1825*	>	2064507/2064570	∼63	No	RNA sequencing	[18]
Teg22as	*Antisens to hypot. protein SA1830*	>	2069004/2069067	∼63	No	RNA sequencing	[18]
Teg124	*Probable β-lactamase/Enterotoxin C3*	<	2059473/2059365	∼108	No	RNA sequencing	[18]
**SaPIn2**							
Sau-63	*Hypot. protein/hypot. protein*	<	436958–437055	∼100	No	Northern	[16]
**SaPIn3**							
sprA1 b	*Trunc. transposase/transposase*	>	1856223–1856978	208	Yes	Northern	[14]
Teg152 b	*transposase/trunc.transposase*	<	1856712–1856658	∼54	No	RNA sequencing	[18]
sprB	Probable*β*-lactamase/truncated HP	<	1866661–1867134	114–118	Yes	Northern	[18]
sprC	*Leukotoxin LukE/Hypot. protein*	<	1871167–1872531	170	Yes	Northern	[14]
**φ (Bacteriophage)**							
sprD	*Hypot. protein/hypot. protein*	<	2006878–2007561	142	Yes	Northern	[37]
sprX(RsaOR)	*Trunc. amidase/staphylokinase*	<	2008572–2009085	147 (processed)	Yes	Northern	[17]
sprF b	*Holin homolog/hypot.protein*	>	2010789–2011001	186	Yes	Northern	[14]
sprG b	*Hypot. protein/holin homolog*	<	2011001–2010789	300	Yes	Northern	[14]

a Except for SprD [37], experimental determinations of the 5′ and 3′ ends were also performed for sprA, sprB, sprC, sprF, sprG, and sprX (unpublished data); the lengths of the RNAs with no ends mapping are approximate;

b RNA couples (underlined) predicted to encode for type I “toxin-antitoxin” modules [18].

RNA candidates Sau-18 and Sau-6079 within SaPIn2, and Sau-6361, Sau-6473, Sau-7007 from φ were predicted but not confirmed by Northern blots [16]. Apart from PIs, *S. aureus* RNAs are also expressed from Genomic Islands (Teg31as and Teg147) and from the staphylococcal chromosome cassette “SCC Mec” (Teg4, Teg5as, Teg6as, Teg7as, Teg8as, Teg10as, Teg14as, Teg118 [18]).

Although the sRNAs expressed from SaPIs are expected to regulate expression of genes located on the cognate PI, they could also establish functional links between the PIs and the core genome. An example is provided by the SprD RNA, expressed from PIφ (Table 1), which represses translation initiation of the *sbi* mRNA encoding an immune-evasion molecule located at a core genomic locus distant from SprD [37]. A central hairpin from SprD pairs to the *sbi* mRNA ribosome binding sites and prevents translation initiation (Figures 1C and 2). Interestingly, SprD contributes significantly to causing disease in a mouse infection model, although this effect is not only linked to the down-regulation of Sbi production. Moreover, overproducing SprD in vivo is toxic for the cells and reduces bacterial growth (S. Chabelskaya, N. Sayed, and B. Felden, unpublished data), possibly suggesting that SprD targets essential function(s). Since SprD has a significant impact on virulence, it implies possible strategies in controlling staphylococcal infections by modulating SprD expression levels. Additional sRNAs expressed from the PIs might also be involved in *S. aureus* pathogenicity, either directly or via intricate regulatory networks including transcriptional regulatory factors.

Among the currently characterized asRNAs expressed in *S. aureus*, four are located in PIs and six in the SCCmec mobile genetic element, all ranging in sizes from 54 to 400 nucleotides [18]. Most of them have perfect base complementarities with regions of mRNAs encoding hypothetical protein genes, and are likely to act as regulators. Two of these sRNAs, Teg152 and SprF, are fully complementary to SprA1 and SprG sRNAs, respectively (Table 1): the “SprA1/Teg152” and “SprG/SprF” RNA pairs are predicted to form type I “toxin-antitoxin” modules in which SprA and SprG would encode hydrophobic small peptides [46]. These modules are found in multiple copies in several *S. aureus* strains, and several copies are expressed (A. Jousselin, M. L. Pinel, and B. Felden, unpublished data). The independent transcriptional activation of the copies allows the production of accurate sRNA levels for precise functions. SprA1 is a multifunctional RNA with putative antisense properties since its 3-end can pair with the 3′-UTRs of three mRNA targets [14]. Interestingly, SCCmec carries determinants other than antibiotic resistance genes, which confer selective advantages to methicillin-resistant *S. aureus* (MRSA) in the host. An sRNA carrying a small ORF was recently identified within the SCCmec. This ORF encodes a peptide that has pro-inflammatory and cytolytic characteristics typical of phenol-soluble modulins (PSM). The PSM-mec peptide had significant impact on immune evasion and disease, thus revealing a role of methicillin resistance clusters in staphylococcal pathogenesis [47]. The expression of the PSM-sRNA (Teg4) was strongly enhanced in response to oxidative stress [18].

## A Multifunctional RNA Couples Quorum Sensing to Virulence

**Figure 3:**
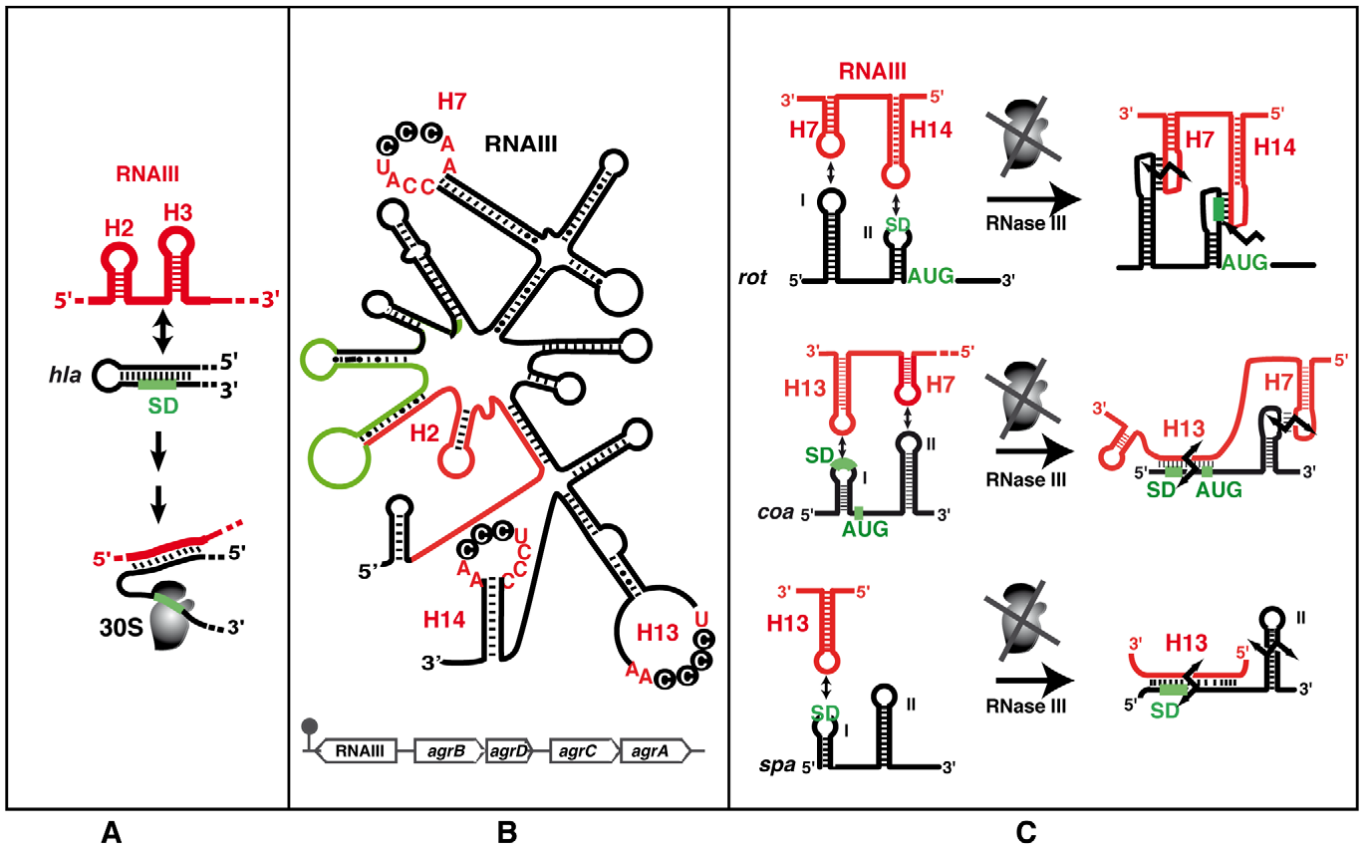
The functional RNAIII structure and its mRNA targets. (A) Schematic view of the RNAIII-mediated antisense activation mechanism. Hairpin loops H2 and H3 of RNAIII (red) bind to the *hla* mRNA (black) to activate translation initiation. The ribosomal 30S subunit is schematized. The SD sequence is green. (B) RNAIII secondary structure (adapted from [52]) and its genomic location within the *agr* locus (bottom). RNAIII encodes the delta-hemolysin (*hld*, in green). The 5′UTR activates alpha-hemolysin translation [54] and the 3′ domain represses the translation of virulence factors and of the transcriptional repressor of toxins (*rot*) [13], [34]. The conserved C-rich sequences detected in H7, H13, and H14 is indicated. (C) Schematic views of the RNAIII-mediated antisense translation initiation repression mechanisms. RNAIII structural domains H7, H13, and H14 (in red) are involved in interactions with target mRNAs (in black). The AUG codon and SD sequence are in green. The broken black arrows are the RNase III–induced cleavages.

RNAIII is the effector of the *agr* system, which functions as a sensor of population density. The complex cascade of events orchestrated by *agr* has been extensively studied (see [4], [12] for review). Briefly, it comprises a density-sensing cassette (*agrD* and *agrB*) and a two-component sensory transduction system (*agrA* and *agrC*) in which autoinducing peptide (AIP), the *agrD* gene product, is continuously released in the extracellular environment. Upon reaching a critical concentration, AIP activates the two-component *agrA*-*agrC* system, which triggers transcription of RNAIII, of its own operon, and of genes encoding metabolic factors and PSM peptides [12], [48]. In this cascade, expression of RNAIII is maximal in the late logarithmic and stationary phase of growth. RNAIII has the unique property of acting both as an mRNA that encodes the 26-aa delta hemolysin (PSM) peptide, and as a critical regulator that represses early virulence factors and activates post-exponentially expressed exotoxins (Figure 3). Further genetic, transcriptomic, and proteomic studies revealed that *agr* belongs to a rich and complex network of regulatory genes in which *agr* is both a target and an effector of regulation (reviewed in [4]). As an effector, RNAIII governs not only the expression of key virulence factors including cell wall–associated proteins and exotoxins, but also numerous two-component systems and global regulators (*arl*, *sae*, *srr*, *rot*) and an impressive list of other processes impacting biofilm formation, peptidoglycan and amino acid metabolism, and transport pathways [49]–[51]. These effects are quantitatively but not qualitatively variable depending on the staphylococcal strain. For instance, the effect of *a*gr inactivation was more marked on the transcriptome of NCTC 8325 derivatives as compared to the UAMS-1 strain [50]. The question as to whether these effects result from direct or indirect mechanisms has been only solved for a limited number of genes and benefited from the experimental determination of RNAIII structure [52]. RNAIII, a highly stable molecule (half-life >45 min) [38] is characterized by 14 stem-loop structures and two long helices formed by long-range base pairings that close off independent structural domains [52]. Specific domains of RNAIII control the expression of different targets (Figure 3). The 5′ end of RNAIII positively regulates *hla* translation (encoding alpha hemolysin) by competing directly with an intramolecular RNA secondary structure that sequesters the *hla* ribosomal binding site (RBS) ([53], [54]; Figure 3A). The RNAIII hairpin H13 and terminator hairpin H14 of the 3′ domain, and hairpin H7 of the central domain, act separately or coordinately to repress the synthesis of early expressed virulence factors (i.e., coagulase, protein A, and the repressor of toxins, Rot) at the post-transcriptional level by a conserved mechanism with slight variations (Figure 3C). The common theme is that RNAIII functions as an asRNA that anneals to target mRNAs, and the formed complexes result in the repression of translation initiation and in rapid mRNA degradation triggered by RNase III. Structures of the complexes depend on the target mRNA, and may comprise i) an extended duplex between RNAIII and the RBS of mRNAs (e.g., *spa*, the peptidoglycan hydrolase LytM, and *SA1000* encoding a fibrinogen-binding protein), or ii) an imperfect duplex that sequesters the RBS completed by a loop–loop interaction in the coding region (for *coa* encoding coagulase), or two loop–loop interactions, one involving the 5′UTR and the other the RBS (for *rot* mRNA) ([13], [39]; Figure 3). In these three cases, a single loop–loop interaction is not sufficient for efficient repression, thus limiting the capacity of RNAIII to act as a repressor to the mRNA targets that not only possess a Shine and Dalgarno (SD) sequence complementary to H7, H13, or H14 of RNAIII, but that also display an additional region of interaction or the capacity to form an extended duplex. As discussed above, the RNA-binding protein Hfq, which is an important RNA chaperone in several species [55], is not involved in the RNAIII-dependent regulatory processes, although Hfq binds to RNAIII in vitro [38].

**Figure 4:**
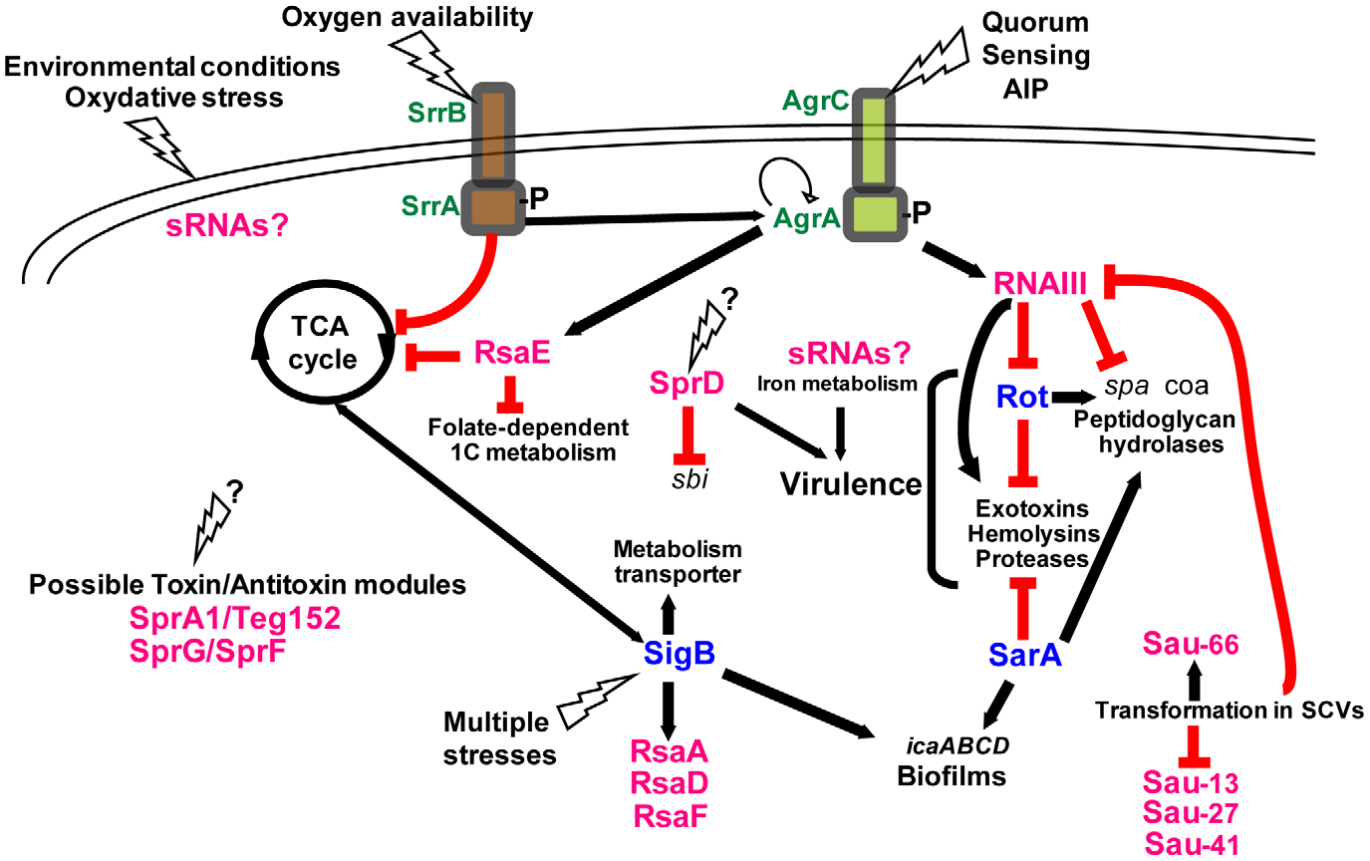
Integrating the *S. aureus* sRNAs into gene regulation cascades. The “*agr*-RNAIII” auto-activation circuit is indicated with the two feed-forward loops involving RNAIII. When reaching optimal density, the autoinducing peptide (AIP) activates the *agr* autocatalytic circuit, leading to RNAIII transcription. RNAIII represses the expression of *rot*, which activates *spa* transcription and represses that of *hla*. In the meantime, RNAIII also activates *hla* mRNA translation and represses *spa* mRNA translation. The plain and broken lines indicate the direct or indirect gene activations, respectively. The red lines indicate the down-regulations mediated by the various RNAs. The black question marks above the “see-sawing” triangles point to the unknown triggering factors. The transcriptional regulatory proteins are in blue. The complexity of this scheme will certainly increase as we learn more on the sRNA functions.

With the exception of *hla* translational activation, all the direct effects of RNAIII lead to repression of mRNA targets. However, as Rot is a transcriptional regulatory protein, its repression by RNAIII results in the indirect transcriptional regulation of many genes, including activation of alpha-toxin and repression of protein A, both of which are also directly regulated by RNAIII [56]. These complex regulatory circuits involve several feed-forward loops (Figure 4) that regulate expression via RNAIII and Rot at both the transcriptional and post-transcriptional levels. For repression, these double controls prevent leakage at the transcriptional level, which could be particularly suitable for stable mRNAs like *spa*. Therefore, the involvement of RNAIII in such regulatory circuits not only guarantees tight regulation but also might ensure fast recovery after the external stimulus (quorum sensing) is over [57]. Hence, a number of genes—and the list will likely grow—are regulated by RNAIII at multiple levels (indirectly on promoter activity, directly on translation and mRNA degradation), suggesting that the amount and timing of production of certain virulence factors is precisely controlled during the course of infection. The in vivo requirement for such strict regulation of virulence protein expression is particularly plausible in the case of the protein A, which harbors multiple functions from anti-opsonic activity to the induction of tumor necrosis factor receptor 1 and B cell superantigenic properties [58], [59].

The importance of *agr* for *S. aureus* pathogenesis is the subject of an apparent paradox. In contrast with most other staphylococcal sRNAs, which have been found by bioinformatic approaches or deep sequencing, RNAIII was first identified in a transposon mutagenesis that revealed pleiotropic effects of a single-site insertion [60], [61]. Because of its impact on virulence factors, the *agr* system and its effector molecule RNAIII were thought to be of major importance for virulence. Indeed, the majorities of clinical isolates from acute infections have a functional *agr* system and produce RNAIII both in vitro and in vivo [62]. However, *agr*-defective mutant strains, which arose during infection, were isolated from patients [62]. Some of these strains have been associated with persistent bacteremia, notably in patients with intravascular devices and with reduced susceptibility to glycopeptides [63], [64]. Agr defects are also detected in colonizing isolates of patients, and a mixture of *agr*-positive and *agr*-defective *S. aureus* strains were described in healthy humans [65]. This supports the model of *agr* being important for full expression of virulence, notably during acute infection, whilst *agr* mutants would be positively selected in chronic infections and dormant states. However, the observation that RNAIII is also present in all staphylococcal species, including *S. epidermidis*, which is typically a nosocomial pathogen, highlights the fact that RNAIII is not solely a determinant of acute virulence but harbors various functions depending on the bacterial species background in which it evolved. Interestingly, the most conserved domain of RNAIII among staphylococcal species is the 3′ domain (H13 and H14), which is involved in the regulation of several *S. aureus*–specific virulence factors (see above). Its conservation in *S. epidermidis, S. lugdunensis*, and other species suggests that some of the target genes, such as those involved in peptidoglycan metabolism, require the presence of this regulatory domain [52], [66].

## *trans*-Acting RNAs in Stress Response and Metabolism

Ongoing functional characterization of *S. aureus* sRNAs links them to various environmental and stress-related responses like pH and temperature variations, nutrient starvation, oxidative stress, and quorum sensing, all of which can be encountered during host infection [15]–[18], [21]. Such environmental stresses and growth conditions largely influence the toxin synthesis [67] and require several global transcriptional regulators, such as the alternative sigma B factor (σ^B^). The σ^B^ regulon consists of numerous genes involved in metabolism, stress-related responses, membrane transport system, biofilm formation, antibiotic resistance, and virulence [68], [69]. Among these genes, several were repressed by σ^B^ via an indirect mechanism most probably involving a σ^B^-induced regulatory protein or sRNA. Along those lines, recent studies showed that the expression of several sRNAs was induced by σ^B^ [15], [70]; among them, RsaA, which has a typical σ^B^-promoter detected upstream of its corresponding gene [15]. RsaA, conserved among staphylococci, can potentially base pair with mRNAs repressed by σ^B^ like *citM* encoding an Mg-citrate transporter (Figure 2; [68]). Prediction of σ^B^-promoter within intergenic regions of the *S. aureus* genome suggests the existence of additional σ^B^-dependent sRNAs, which awaits experimental validation [15], [18]. Three σ^B^-dependent sRNAs that are highly conserved in *S. aureus* have been recently described [71], and two of them are predicted to encode small highly basic peptides [18], [71].

Most of the newly identified sRNAs are conserved among *S. aureus* clinical isolates or are expressed in various staphylococcal strains [14], [15], [17], [18]. One exception is the sRNA RsaE for which the sequence and structure have been found strictly conserved in the *Staphylococcus*, *Macrococcus*, and *Bacillus* genera, all of which share a common Gram-positive ancestor (Figure 2; [15]). The overproduction of RsaE causes a growth defect that is partially alleviated by the non-preferred carbon source, acetate, suggesting that RsaE accumulation alters essential metabolic functions [17]. Using comparative transcriptomic and proteomic analysis, RsaE was shown to co-regulate the synthesis of several metabolic pathways involved in amino acid and peptide transport (*opp-3* operon), cofactor synthesis, folate-dependent one-carbon metabolism, lipid and carbohydrate metabolism, and the tricarboxylic acid (TCA) cycle [15], [17]. Like RNAIII, a conserved and unpaired C-rich motif within RsaE pairs with the SD sequence of several target mRNAs, including *opp-3B/opp-3A* (amino acid and peptide transporter), *sucC* (succinyl-CoA synthetase of the TCA cycle), and SA0873 (unknown function), all preventing ribosomal initiation complex formation (Figure 2; [15], [17]). Thus, RsaE would coordinate down-regulation of energy metabolism (via the TCA cycle) and purine biosynthesis when carbon sources become scarce, facilitating adaptation to the entry into stationary phase (Figure 4). The TCA cycle is integrally involved in the regulation of virulence factor synthesis, biofilm formation and antibiotic resistance (for a review, see [67]). Other sRNAs are probably involved in metabolic regulation (Figure 4). For instance, carbohydrate-dependent repression and oxygen availability correlate with altered expression of RNAIII [67]. Although no iron-dependent sRNA was so far identified in *S. aureus* as in Gram-negative bacteria [65], *S. aureus* sense the alteration of iron status via the ferric uptake regulator (Fur), initiating a regulatory program that modifies expression of a large number of virulence factors [72]. The ongoing functional analysis of the *S. aureus* sRNAs will provide a clearer picture of the links between sRNAs, metabolism, stress adaptation, and virulence programming.

## RNAs as Antimicrobial Drug Targets

The continued evolution of anti-microbial resistance in hospitals and the emergence of community-associated MRSA strains are major threats to patient care. Current antibiotic drugs target a narrow spectrum of bacterial functions including peptidoglycan biogenesis, DNA replication, and protein synthesis. Therefore, there is a growing need for selecting new drugs that target other cellular pathways that should, in principle, result in a weaker selective pressure for the appearance of antibiotic resistance, and that can preserve the host endogenous microbiome. As alternative strategies that affect bacterial viability, anti-virulence strategies have been developed to target mechanisms leading to successful infections, such as virulence factors causing host damage and disease [73]. Among all the antibiotics currently used to treat clinical infections, more than half bind to the ribosomal RNAs [74]. Their success as antibacterial targets encourages the development of new antibacterial drugs based on regulatory structured sRNAs. Metabolite-sensing mRNAs, the so-called riboswitches, have been recently exploited as drug targets since they have evolved structured receptors to bind small metabolites with high selectivity and to control the expression of downstream essential genes [75].

Riboswitches are located in the 5′UTRs of some mRNAs and exhibit a structured receptor domain specifically recognized by a small compound. Metabolite binding induces a conformational change of the downstream mRNA that provokes either premature transcription, translation repression, or RNA degradation. In *S. aureus*, seven operons and 33 genes are under the control of riboswitches that respond to the intracellular concentration of S-adenosylmethionine (SAM) (Figure 1A), thiamine pyrophosphate (TPP), flavin mononucleotide (FMN), lysine, glycine, guanine, 7-aminomethyl-7-deazaguanine (preQ1), and glucosamine-6-phosphate (Glc-6P) [15], [17], [18], [20], [75]. Any agonistic molecule targeting one of these riboswitches would likely impact gene regulation even if cells are devoid of the natural metabolite. As a proof of principle, and based on the crystal structure of the guanine receptor binding domain [76], [77], several rationally designed guanine analogues that bind the purine riboswitch with affinities comparable to that of the natural ligand were shown to inhibit *B. subtilis* growth [78]. In *S. aureus*, the guanine riboswitch regulates expression of the operon, including *xpt*, *pbuX*, *guaB*, and *guaA*. Using the same strategy as Kim et al. [78], a novel pyrimidine derivative, 2,5,6-triaminopyrimidin-4-one (PC1), was designed to bind the purine-sensing riboswitch to repress the downstream genes [79]. For the first time, this work shows that PC1 has a selective bactericidal activity restricted to a sub-group of bacteria including *S. aureus*, which contains *guaA* under the control of the purine riboswitch. Although the GMP synthase GuaA is not essential for growth in rich media, the enzyme is nevertheless an important contributor to *S. aureus* survival during infection [79]. The administration of PC1 significantly reduced *S. aureus* infection in a murine model [79]. The narrow spectrum of bactericidal activity of PC1 also has the advantage of reducing the selective pressure for resistance. This work and the fact that *S. aureus* contains other types of riboswitches offer novel opportunities for the design of drugs that inhibit the function of structured regulatory RNAs. The increasing rate of *S. aureus* sRNA discovery, together with the intensified search for their mechanisms of action, should pave the way to exploit chemical strategies to interfere with sRNA functions and to fight against bacterial infections in a more specific way.

## Concluding Remarks

This review provides a first hint at sRNA functions in *S. aureus* and shows that we are just beginning to fully appreciate their roles in gene regulation. The combined use of high throughput genomic methods and phenotypic analyses of *S. aureus* strains mutated for the sRNA genes, regulatory proteins, ribonucleases, and RNA-binding proteins will generate knowledge on how the regulatory RNAs and proteins are integrated into intertwined regulatory networks in stress adaptation and virulence (Figure 4). However, complications are expected due to the substantial genetic variability between *S. aureus* strains, which express a subset of regulatory RNAs, or unique RNAs, and are thus far from universal [14], [15].

Future research is also necessary to identify the signals that regulate sRNA transcription and the mechanisms by which sRNAs act on their targets. To date, most identified mechanisms have involved *trans*-acting sRNAs that bind to the RBS of mRNA targets, and only SprA was predicted to bind to the 3′UTRs. Binding to the coding sequence has not yet been observed. While scientific interest has been mainly focused on antisense regulation, regulatory RNAs are also expected to target proteins. For instance, direct interaction of *S. aureus* 6S RNA with the polymerase bound to σ^A^ and its implication in virulence needs to be analyzed. Multifunctional RNAs, like RNAIII, are most probably the rule rather than the exception, and this field is at present completely unexplored. We also need to consider other unexpected possibilities such as RNA-activating virulence factors, or bacterial sRNAs targeting host genes. Recent analysis of the MRSA operon also shows that many mRNAs have long UTRs, more frequently found at the 3′ ends [80]. These regions might have implications in regulation by promoting specific binding sites for *trans*-acting ligands or by their processing to generate sRNAs [18]. Mechanisms of RNA processing and turnover are not well studied in *S. aureus* and little is known about *S. aureus* RNA-binding proteins associated with sRNAs.

This review illustrates the great diversity in sizes, structures, and mechanisms of sRNAs, and shows that determinants required for regulation could sometimes be predicted from the RNA structure. For instance, several sRNAs and RNAIII carry a C-rich motif, located in hairpin loops or in accessible single strands, which is a specific recognition signature to target the mRNA RBS [13], [15]. Determination of the structures of regulatory complexes has paved the way to identify novel drugs that could interfere with RNA functions [79]. Also, the significant contribution of RNAIII and SprD to cause diseases in animal models of infection implies that these RNAs could be promising drug targets.

Another aspect, which might be important for virulence and host adaptation, would be to consider the cell differentiation within a population, as well as cell-to-cell communication. The expression of individual sRNAs might be variable within a population and these differences could confer the ability of a bacterial subpopulation to respond to stress or environmental changes. This is particularly true for biofilm formation. Furthermore, we have no idea how the host “microbiome” influences sRNA expression and their regulatory networks within *S. aureus*, and vice versa. Metagenomics and deep sequencing could address these questions and would contribute to an understanding of how commensal bacteria can cause diseases.

The recently identified *S. aureus* RNome (listed in Table S1) reveals additional layers of complexity to gene regulation mechanisms. sRNA-based regulation increases the number of possible regulatory sites and expectedly provides several advantages compared to protein-based regulation [81]. Since many sRNAs act at transcription termination or when translation starts, fast and efficient responses on protein levels can be achieved. Furthermore, it is also easier to control RNA turnover when compared to protein degradation. To date, the known *S. aureus* regulatory RNAs provide functional links between metabolism, quorum sensing, and virulence (Figure 4). As we learn more about sRNA functions, we expect to find more sRNAs involved in *S. aureus* pathogenesis as well as other connections between virulence and housekeeping networks.

## Supporting Information

Table S1 Compilation of 91 expressed RNAs forming the *S. aureus* RNome. It includes 18 *cis*-acting regulators including riboswitches (green), 7 *cis*-encoded sRNAs (purple) 47 *trans*-encoded RNAs (pink), 10 repeated sequences (yellow), and 9 potential 5′UTRs, 3′UTRs, or small CDSs. Both the gene and RNA names, their experimental confirmations, strand expression, genomic locations, predicted lengths, flanking and/or antisense genes, expression profiles, and conservations are indicated for each RNA with specific comments about their functions, when available.
(XLS)
